# MIF/SCL3A2 depletion inhibits the proliferation and metastasis of colorectal cancer cells via the AKT/GSK‐3β pathway and cell iron death

**DOI:** 10.1111/jcmm.17352

**Published:** 2022-05-13

**Authors:** Guan Huang, Lili Ma, Lan Shen, Yan Lei, Lili Guo, Yongjian Deng, Yanqing Ding

**Affiliations:** ^1^ Department of Pathology Nanfang Hospital and School of Basic Medical Sciences Southern Medical University Guangzhou China; ^2^ Department of Pathology Shenzhen Longgang Central Hospital Shenzhen China; ^3^ Guangdong Provincial Key Laboratory of Molecular Tumor Pathology Guangzhou China

**Keywords:** Akt/GSK‐3β signalling, colorectal cancer, macrophage migration inhibitory factor, SLC3A2

## Abstract

This study investigated the mechanisms of migration inhibitory factor (MIF) and solute carrier family 3 member 2 (SLC3A2) in colorectal cancer progression. The levels of MIF and SLC3A2 expression in cells were measured by RT‐qPCR. SW480 and SW620 cells were transfected with sh‐MIF and sh‐SLC3A2, respectively. MIF, SLC3A2, GPX4, E‐cadherin and N‐cadherin expression were detected by immunofluorescence (IF). CCK8 and Transwell assays were performed to detect cell proliferation and migration. Co‐immunoprecipitation (CoIP) was used to measure the binding activity of MIF and SLC3A2. Finally, a nude mouse tumorigenicity assay was used to confirm the functions of MIF and SLC3A2 in colorectal cancer. Results showed that the levels of MIF and SLC3A2 expression were up‐regulated in colorectal cancer cells. Inhibition of MIF or SLC3A2 expression prevented cell proliferation, migration, epithelial‐mesenchymal transition (EMT) and invasion. In addition, knockdown of MIF and SLC3A2 promoted iron death in SW480 and SW620 cells. CoIP results showed that MIF and SLC3A2 directly interact with each other. Knockdown of both MIF and SLC3A2 inhibited tumour growth and metastasis via the AKT/GSK‐3β pathway *in vivo*. The Akt/GSK‐3β pathway was found to participate in regulating MIF and SLC3A2 both in vivo and in vitro. MIF and SLC3A2 might be potential biomarkers for monitoring the treatment of colorectal cancer.

## 1 INTRODUCTION

Colorectal cancer (CRC) is a malignant cancer occurring in the colon or rectum.^1^ Rectal cancer is more common than colon cancer.^2^ According to statistics, colorectal cancer is more common in adults >40 years of age and occurs more often males than in females.^3^ The incidence and mortality rates of colorectal cancer rank fifth in the world among all malignant tumours.^4^

Macrophage migration inhibitory factor (MIF) is a highly conserved protein with a size of 12.5 Kd; it was first discovered and reported in lymphoid tissue in 1932.^5^ MIF can be produced by a variety of cells, including lymphocytes, neutrophils and mast cells.^6^ MIF is an immune cytokine that is expressed at low levels in various epithelial cells, and participates in the pathogenesis of inflammatory and immune diseases.^7^ Increasing evidence suggests that abnormal MIF expression is closely associated with different inflammatory diseases and certain cancers.^8^, ^9^ It has been reported that MIF levels are increased in gastric cancer, and regulate lncZFPM2‐AS1 to promote the development of gastric cancer.^10^ However, the role played by MIF in intestinal tumours requires further investigation.

SLC3A2 (Solute carrier family 3 member 2), also known as CD98hc, is an important member of the SLC family.^11^ SLC3A2 is a transmembrane dimer protein that regulates intracellular signal transduction by binding to integrin proteins.^12^ Recent studies have found that SLC3A2 is up‐regulated in a variety of tumour tissues.^13^ In addition, SLC3A2 participates in regulating multiple biological pathways, including the AKT/GSK3β signalling pathway.^14^ AKT, a protein kinase, is involved in apoptosis and glucose metabolism, and activates downstream GSK‐3β.^15^ Studies have shown that the Akt/GSK‐3β signalling pathway plays an important role in the occurrence of various cancers.^16^, ^17^ Epithelial‐mesenchymal transition (EMT) is believed to lead to a loss of adhesion between tumour cells during the malignant progression of tumours, resulting in enhanced cell invasion and mobility. The AKT/GSK‐3β pathway is closely related to the EMT process and participates in various biological and pathological processes, such as tumour cell invasion and metastasis.

Iron death is a type of iron‐dependent cell death that differs from apoptosis, necrosis and autophagy. Iron death leads to smaller mitochondria, higher membrane density and fewer cristae.^18^ Iron death is characterized by increased lipid peroxidation and ROS levels.^19^ The main mechanism of iron death is GPx4 inactivation caused by GSH consumption. The GPx4 enzyme changes the peroxy bond of a peroxidized lipid into a hydroxyl group, which causes the lipid to lose its peroxidation activity.^20^ Recent studies have shown that iron death is related to the development and progression of cancer.^21^ Dixon et al.^22^ revealed the important role played by the ACSl4 enzyme in cell death and suggested stimulation of cell iron death as a new strategy for treating human cancers. Therefore, further study of the mechanism of iron death is of great significance for finding new tumour targets and targeted drugs.

Our study revealed that the levels of both MIF and SLC3A2 expression were increased in SW480 and SW620 cells, and a downregulation of those expression levels inhibited colorectal cancer cell proliferation, promoted cell iron death, inhibited cell migration and the EMT process, and affected the AKT/GSK‐3β signalling pathway. Further experiments showed that knockdown of MIF and SLC3A2 inhibited tumour growth and metastasis by promoting iron death and regulating the AKT/GSK‐3β pathway *in vivo*.

## 2 MATERIALS AND METHODS

### 2.1 Cell lines

Human colorectal cancer cell lines SW480, SW620, HCT116, HT‐29 and LoVo, as well as colon epithelial NCM460 and FHC cells were purchased from the American Type Culture Collection (Manassas, VA, USA). All the above cell lines were cultured in Dulbecco's modified Eagle's medium (DMEM) supplemented with 10% FBS (foetal bovine serum; Thermo Fisher Scientific) and 100 U/ml penicillin/streptomycin (Sigma‐Aldrich) at 37°C in a humidified thermal incubator containing 5% CO_2_.

### 2.2 Animals

A total of 20 female BALB/c nude mice (age, 5–7 weeks; weight ~20 g) were housed in a laboratory facility maintained at 22°C, 55% relative humidity and a 12 light/dark cycle. Sterilized special feed was provided, and food and water were available ad libitum. After one week of feeding, the mice were assigned to five separate groups. The protocols for all animal experiments were approved by the local Ethics Committee. SW480 cells from the control, SHNC, shMIF, shSLC3A2 and shMIF + shSLC3A2 groups were grown to 80%–90% confluence; after which, they were digested with trypsin (1 ml) and resuspended. The number of cells in each sample was adjusted to ~2 × 10^7^ cells/ml. After disinfection, 200 μl of suspended cells was injected into the right armpit of each mouse via subcutaneous injection on the back. The sizes of the resultant tumours were recorded.

### 2.3 Transfection

The sequences of shMIF and shSLC3A2 were synthesized (Sangon Biotech) and transfected into SW480 and SW620 cells by using Lipofectamine 3000 reagent (#L3000001; Thermo Fisher Scientific) according to the manufacturer's instructions. The sequence with the highest efficiency for gene silencing was selected as the Small hairpin RNA (shMIF and shSLC3A2) and ligated with the lenti‐virus LV003 vector. A negative control sequence was used as the Scramble (shNC) sequence. The above vectors were used for transfecting HEK293T cells together with packaging vectors in the preparation of recombinant virus vectors, which were then transfected into SW480 and SW620 cells as described above. Stable clones for MIF and SLC3A2 shRNA‐expressing cells were generated by using the lentiviral vectors and selection in puromycin.

### 2.4 Cell Counting Kit (CCK‐8) assay

SW480 and SW620 cells were seeded into the wells of 96‐well plates. After overnight culture, the cells were exposed to gemcitabine at the designed concentrations, and cell viability was measured at 12 h or 24 h later. CCK‐8 reagent (10 μl; Beyotime) was added to each well, and after one hour of incubation, the optical density of each well at 450 nm was determined with a spectrophotometer (Bio‐Rad).

### 2.5 Transwell assay

For detection of cancer cell migration, the upper chambers of Transwell plates were filled with serum‐free DMEM supplemented with penicillin/streptomycin (1%), while the lower chambers contained complete DMEM supplemented with FBS (10%) and penicillin/streptomycin (1%). Cancer cells were seeded into the upper chambers and incubated under normal culture conditions for 2 days; after which, the cancer cells that had migrated into the lower chambers were stained with 0.1% crystal violet for 10 min, and then counted and photographed under a microscope. To assess cell invasion, the cells were analysed using the same procedures as described above, except that the inner sides of the Transwell plates were first coated with Matrigel (BD Biosciences).

### 2.6 Determination of ROS

The cells in each group were collected and washed twice with cold PBS. Next, 2 μl of trypsin was added to each group of cells, and the cells were centrifuged for 5 min. After removing the supernatants, the cells were treated with an ROS probe (10 μM carboxy 2’‐7’‐dichlorofluorescein diacetate; cat C400; Molecular Probes, Thermo Fisher Scientific) in PBS at room temperature for 30 min. Next, the cells were harvested by trypsinization, resuspended in PBS containing 2% FBS and analysed by flow cytometry (BD FacsVerse, BD Biosciences). The percentage of positive events in the FL1 (FITC) channel (ROS‐producing cells) was recorded. The above experiment was repeated three times, and the average value was recorded.

### 2.7 H&E staining

The histological characteristics of tumours in nude mice were evaluated by haematoxylin and eosin (H&E) staining. Briefly, tumour tissue sections (5 μm thick) were fixed with 4% neutral phosphate‐buffered formalin at 4°C, embedded in paraffin and subjected to staining by using a Hematoxylin‐Eosin staining kit (Sangon Biotech) according to the manufacturer's instructions.

### 2.8 Immunohistochemical staining

Specimens of tumour tissue were fixed with 4% paraformaldehyde at 37˚C for 48 h. Formalin was used to fix the paraffin‐embedded specimens for immunohistochemical staining, which performed as described by Hou et al.^23^ The antibodies used for immunohistochemical staining were as follows: Ki‐67 (1:800), E‐cadherin (1:1000), N‐cadherin (1:800) and GPX4 (1:1000, Abcam). Tissue samples were incubated with the primary polyclonal antibody overnight at 4°C; after which, they were incubated with a biotinylated goat anti‐mouse IgG antibody (1:1,000; cat. no. AP124; Sigma‐Aldrich; Merck KGaA) at 37˚C for 30 min. Images were captured with an inverted fluorescence microscope (Nikon Ni‐U; Nikon Corporation; magnification, 200×).

### 2.9 RT‐qPCR

RT‐qPCR was performed as described in a previous study.^24^ In brief, the total RNA was extracted from cells using Trizol Reagent (Ambion) and then quantified by spectrophotometry. Next, the total RNA was reverse transcribed into cDNA using Primescript RT Reagents (Life Technologies) according to the manufacturer's protocol. RT‐PCR was performed in triplicate by using a GoTaq^®^ qPCR Master Mix kit (Promega) on an ABI 7500 Real‐time PCR System (Bio‐Rad). The reaction protocol was as follows: initial denaturation at 95°C for 1 min, followed by 35 cycles of denaturation at 95°C for 30 s, annealing at 62°C for 30 s and elongation at 72°C for 30 s. GAPDH served as an internal control, and relative levels of gene expression were determined by the 2^−ΔΔCT^ method. The specific primer sequences used were as follows: GAPDH: F, 5′‐TGTTCGTCATGGGTGTGAAC‐3′ and R, 5′‐ATGGCATGGACTGTGGTCAT‐3′; E‐cadherin: F, 5′‐ATTTTTCCCTCGACACCCGAT‐3′ and R, 5′‐TCCCAGGCGTAGACCAAGA‐3′; N‐cadherin: F, 5′‐TGCGGTACAGTGTAACTGGG‐3′ and R,5′‐GAAACCGGGCTATCTGCTCG‐3′; MIF: F,5′‐GAACCGCTCCTACAGCAAG‐3′ and R, 5′‐AGTTGTTCCAGCCCACATTG‐3′; SLC3A2: F, 5′‐CAACTACCGGGGTGAGAACT‐3′ and R, 5′‐TATGTCCCGAACCTGGAACC‐3′; GPX4: F,5′‐GAGGCAAGACCGAAGTAAACTAC‐3′ and R, 5′‐CCGAACTGGTTACACGGGAA‐3′.

### 2.10 Western blotting

The total proteins were extracted from cultured cell lines or mouse tissues by using a Total Protein Extraction kit (KeyGen Biotech) according to the manufacturer's protocol. The amount of total protein in each extract was determined using the BCA method. Next, a 30 ug sample of total protein from each extract was boiled in protein loading buffer at 100°C and then separated by 15% SDS‐PAGE. The separated protein bands were transferred onto PVDF membranes (Millipore), which were subsequently blocked with lipid‐free milk solution (5%) for 2 h at room temperature. The membranes were then incubated with diluted primary and secondary antibodies, and the immunostained protein bands were visualized using highly sensitive ECL (enhanced chemiluminescence) reagent (Abcam). GAPDH served as an internal standard. The primary antibodies used for Western blotting were anti‐MIF (1:500), anti‐SLC3A2 (1:800), anti‐GPX4 (1:800), anti‐pAKT (1:800), anti‐pGSK‐3β (1:1000), anti‐E‐cadherin (1:1000) and anti‐N‐cadherin (1:800).

### 2.11 Statistical analysis

All data were analysed using SPSS for Windows, Version 13.0 (SPSS Inc.), and results are expressed as the mean value ± SD of data obtained from at least three independent experiments. The statistical significance of differences between groups was assessed by the Student's *t*‐test. A *p*‐value < 0.05 was considered to be statistically significant.

## 3 RESULTS

### 3.1 MIF and SLC3A2 were more highly expressed in colorectal cancer cells than in normal intestinal epithelial cells

**FIGURE 1:**
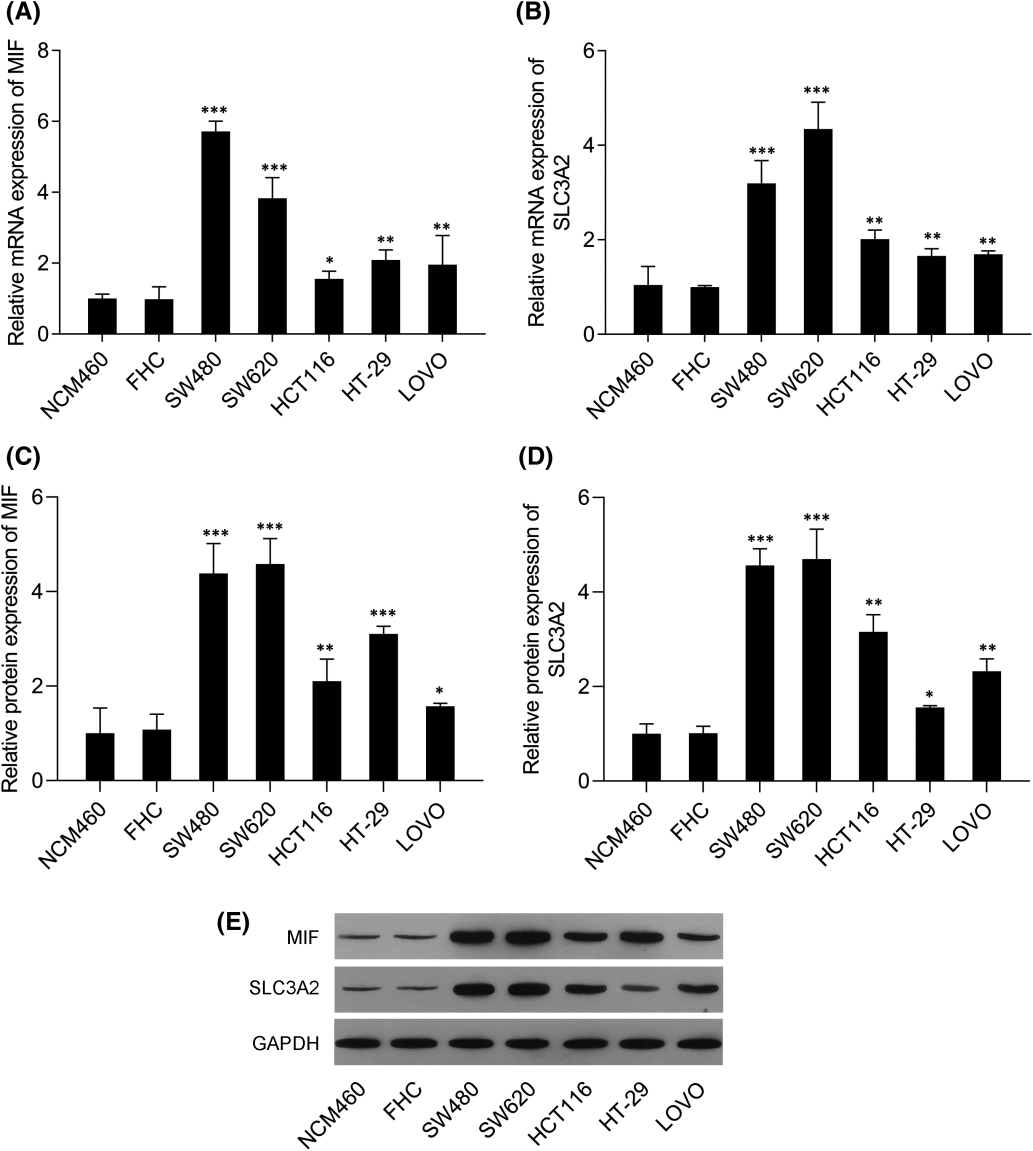
Levels of MIF and SLC3A2 expression in colorectal cancer cells were higher than those in normal intestinal epithelial cells. (A) MIF mRNA expression in NCM460, FHC, SW480, SW620, HCT116, HT‐29 and LoVo cells. (B) SLC3A2 mRNA expression in NCM460, FHC, SW480, SW620, HCT116, HT‐29 and LoVo cells. (C) MIF protein expression in NCM460, FHC, SW480, SW620, HCT116, HT‐29 and LoVo cells. (D) SLC3A2 protein expression in NCM460, FHC, SW480, SW620, HCT116, HT‐29 and LoVo cells. (E) Western blotting was performed to confirm the levels of MIF and SLC3A2 protein expression in NCM460, FHC, SW480, SW620, HCT116, HT‐29 and LoVo cells. ‘*’ means *p *< 0.05 when compared with the NCM460 or FHC group; ‘**’ means *p* < 0.01 when compared with the NCM460 or FHC group; ‘***’ means *p* < 0.001 when compared with the NCM460 or FHC group. GAPDH served as an invariant internal control for calculating fold changes in protein expression

We found that the levels of MIF and SLC3A2 mRNA and protein expression were notably increased in the colorectal cancer cells (SW480, SW620, HCT116, HT‐29 and LoVo) when compared with normal epithelial cells (NCM460, FHC) (Figure 1A–E). SW480 and SW620 cells were selected for use in subsequent experiments due to their high levels of MIF expression.

### 3.2 Knockdown of MIF and SLC3A2 expression in SW480 and SW620 cells

**FIGURE 2:**
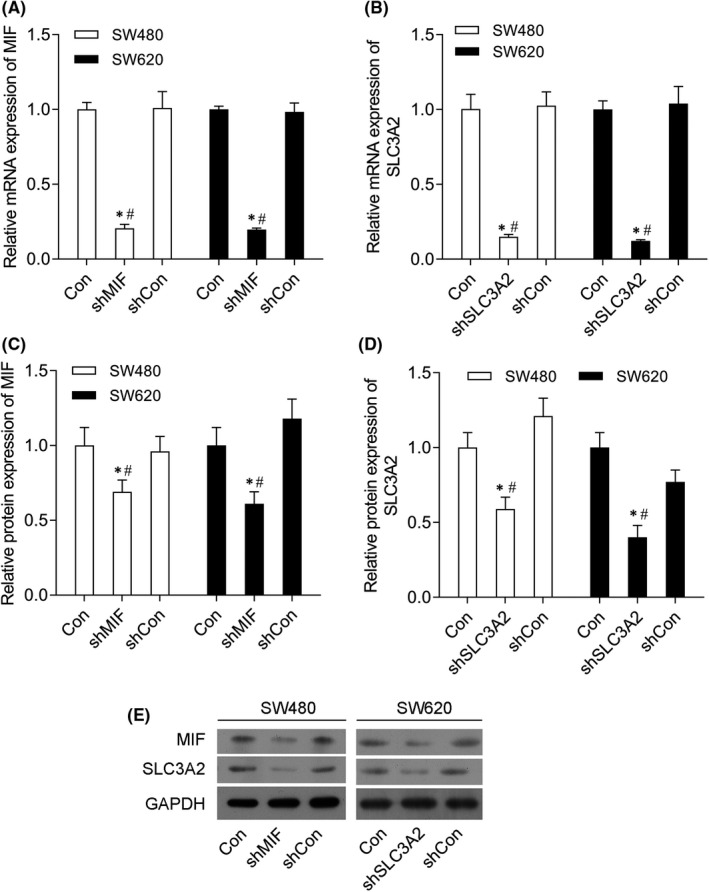
Knockdown of MIF and SLC3A2 inhibited MIF and SLC3A2 expression in SW480 and SW620 cells. SW480 and SW620 cells were transfected with control shRNA (shCon group), shMIF (shMIF group), shSLC3A2 (shSLC3A2 group) or co‐transfected with shMIF plus shSLC3A2 (shMIF+shSLC3A2 group), respectively. (A) MIF mRNA expression in SW480 and SW620 cells in the control, shMIF and shCon groups. (B) SLC3A2 mRNA expression in SW480 and SW620 cells in the control, shSLC3A2 and shCon groups. (C) MIF protein expression in SW480 and SW620 cells in the control, shMIF and shCon groups. (D) SLC3A2 protein expression in SW480 and SW620 cells in the control, shSLC3A2 and shCon groups. (E) Western blotting was performed to confirm the levels of MIF and SLC3A2 protein expression in SW480 and SW620 cells. ‘*’ means *p *< 0.05 when compared with the control group; ‘^#^’ means *p* < 0.05 when compared with the shCon group. GAPDH served as an invariant internal control for calculating fold changes in protein expression

The levels of MIF mRNA and protein expression in SW480 and SW620 cells in the shMIF groups were notable decreased when compared with those in the shCon and control groups of SW480 and SW620 cells (Figure 2A, C and E). In addition, SLC3A2 expression was also notably decreased in SW480 and SW620 cells transfected with shSLC3A2 (Figure 2B, D and E).

### 3.3 Knockdown of MIF and SLC3A2 expression inhibited cell proliferation and migration

**FIGURE 3:**
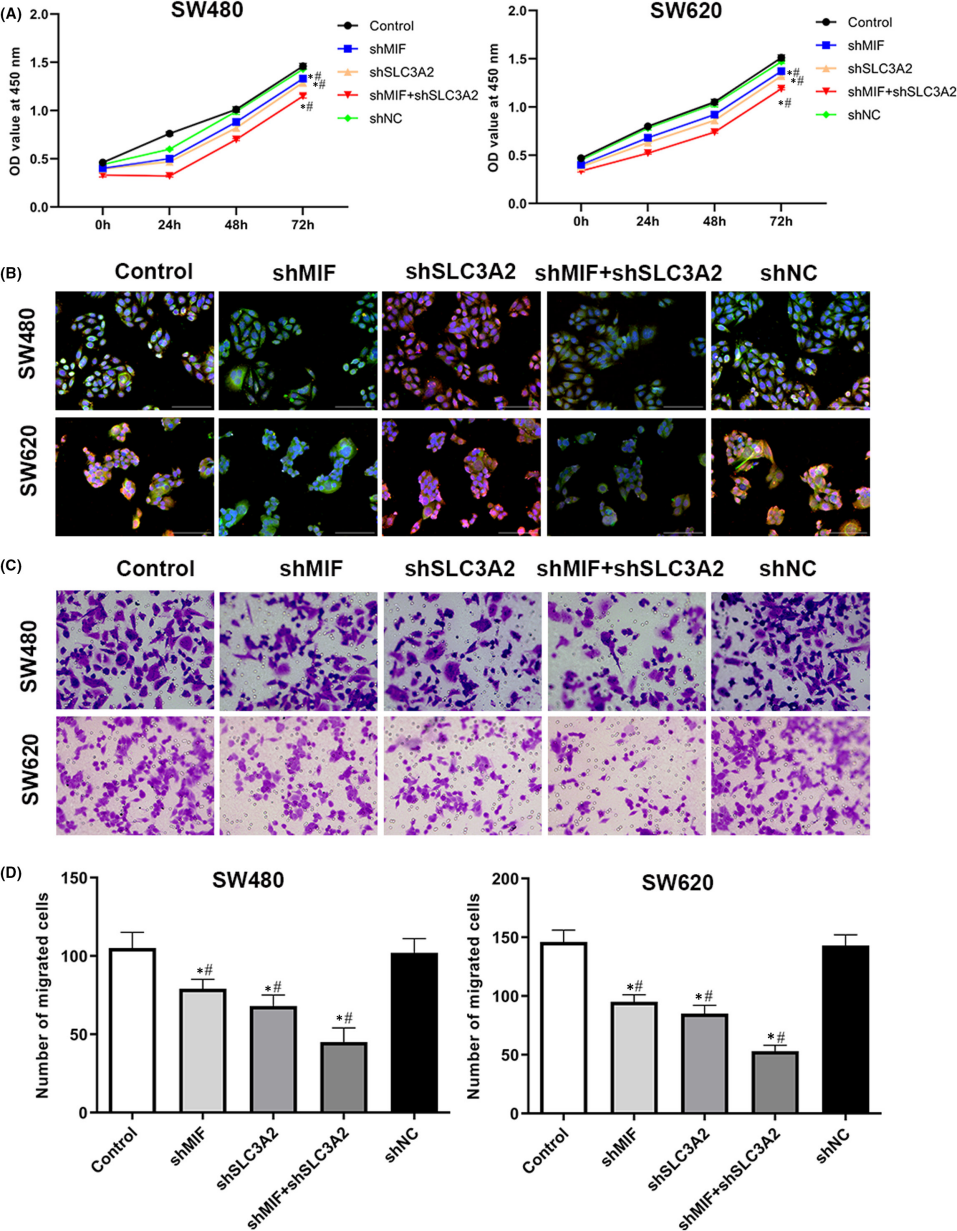
Knockdown of MIF and SLC3A2 inhibited the proliferation and migration of SW480 and SW620 cells. (A) The OD values of SW480 cells at 450 nm. (B) Immunofluorescence assays were performed to detect MIF and SLC3A2 expression in SW480 and SW620 cells. (C–D) Transwell assays were performed to measure the migration of SW480 and SW620 cells. ‘*’ means *p* < 0.05 when compared with the control group; ‘#’ means *p* < 0.05 when compared with the shCon group. GAPDH served as an invariant internal control for calculating fold changes in protein expression

CCK‐8 assay results showed that cell proliferation was significantly reduced in cells transfected with shMIF or shSLC3A2, and that trend was even more significant in cells that had been co‐co‐transfected with shMIF plus shSLC3A2 (Figure 3A). In addition, immunofluorescence assays showed that the levels of MIF and SLC3A2 expression were notably decreased in the shMIF and shSLC3A2 groups, and were even lower in the co‐transfection groups (Figure 3B). Transwell assay results showed that shMIF and shSLC3A2 significantly inhibited cell migration (Figure 3C, D).

### 3.4 Knockdown of MIF and SLC3A2 expression inhibited the epithelial‐mesenchymal transition (EMT) process

**FIGURE 4:**
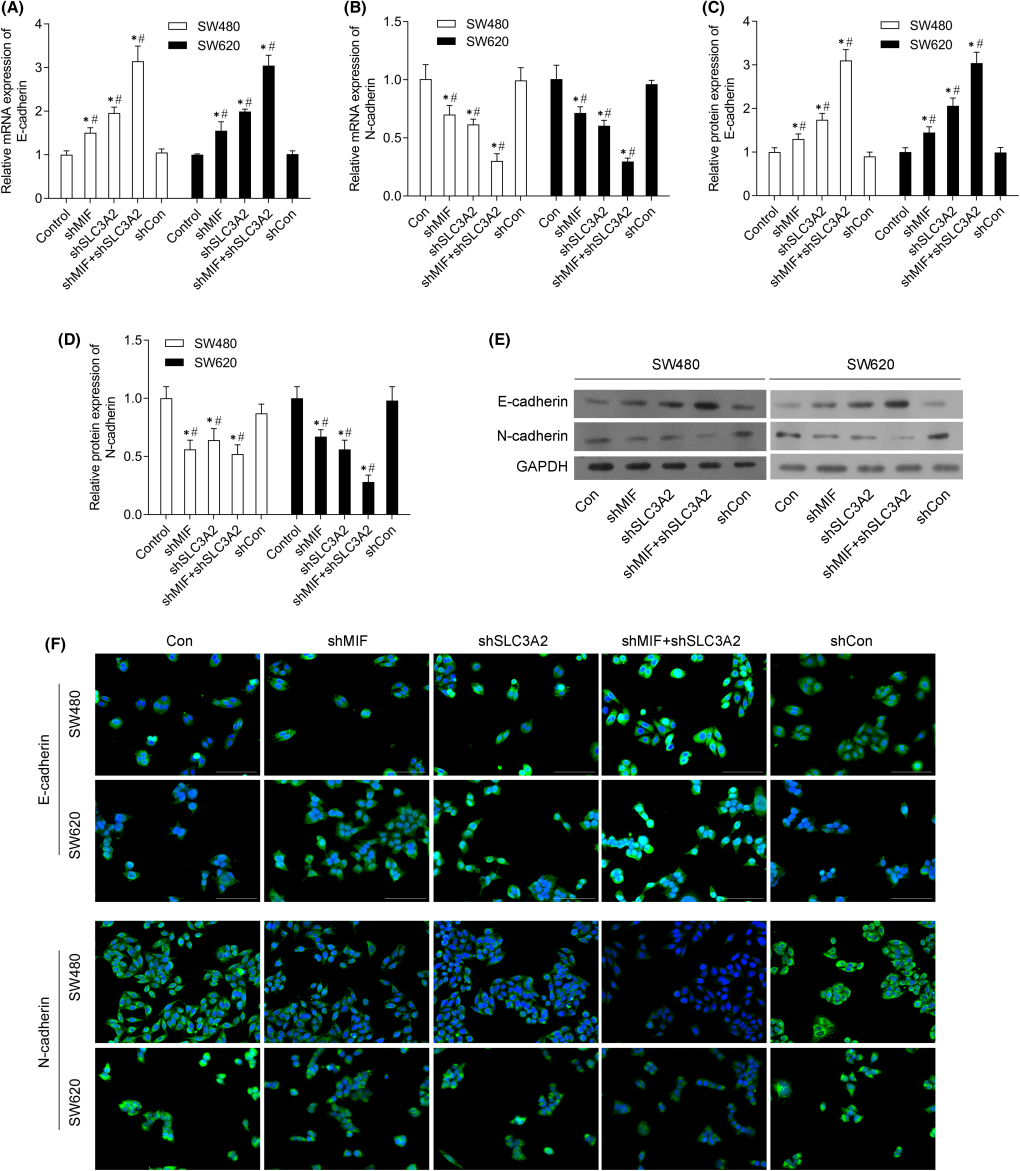
Knockdown of MIF and SLC3A2 inhibited epithelial‐mesenchymal transition (EMT) in SW480 and SW620 cells. SW480 and SW620 cells were transfected with control shRNA (shCon group), shMIF (shMIF group), shSLC3A2 (shSLC3A2 group) or co‐transfected with shMIF plus shSLC3A2 (shMIF + shSLC3A2 group), respectively. (A) E‐cadherin mRNA expression in SW480 and SW620 cells. (B) N‐cadherin mRNA expression in SW480 and SW620 cells. (C) E‐cadherin protein expression in SW480 and SW620 cells. (D) E‐cadherin protein expression in SW480 and SW620 cells. (E) Western blotting was performed to confirm the levels of E‐cadherin and N‐cadherin protein expression in SW480 and SW620 cells. (F) Immunofluorescence assays were performed to detect E‐cadherin and N‐cadherin protein expression in SW480 and SW620 cells. ‘*’means *p *< 0.05 when compared with the control group; ‘^#^’ means *p* < 0.05 when compared with shCon group. GAPDH served as an invariant internal control for calculating fold changes in protein expression

We found that shMIF and shSLC3A2 significantly promoted E‐cadherin mRNA and protein expression (Figure 4A, C and E), but inhibited N‐cadherin mRNA and protein expression (Figure 4B, D and E). In addition, immunofluorescence assays showed that E‐cadherin expression was notably increased in the shMIF and shSLC3A2 groups, and was even higher in the shMIF + shSLC3A2 group, while N‐cadherin expression was decreased in the shMIF and shSLC3A2 groups, and was even lower in the shMIF + shSLC3A2 group (Figure 4F).

### 3.5 Knockdown of MIF and SLC3A2 expression promoted iron death

**FIGURE 5:**
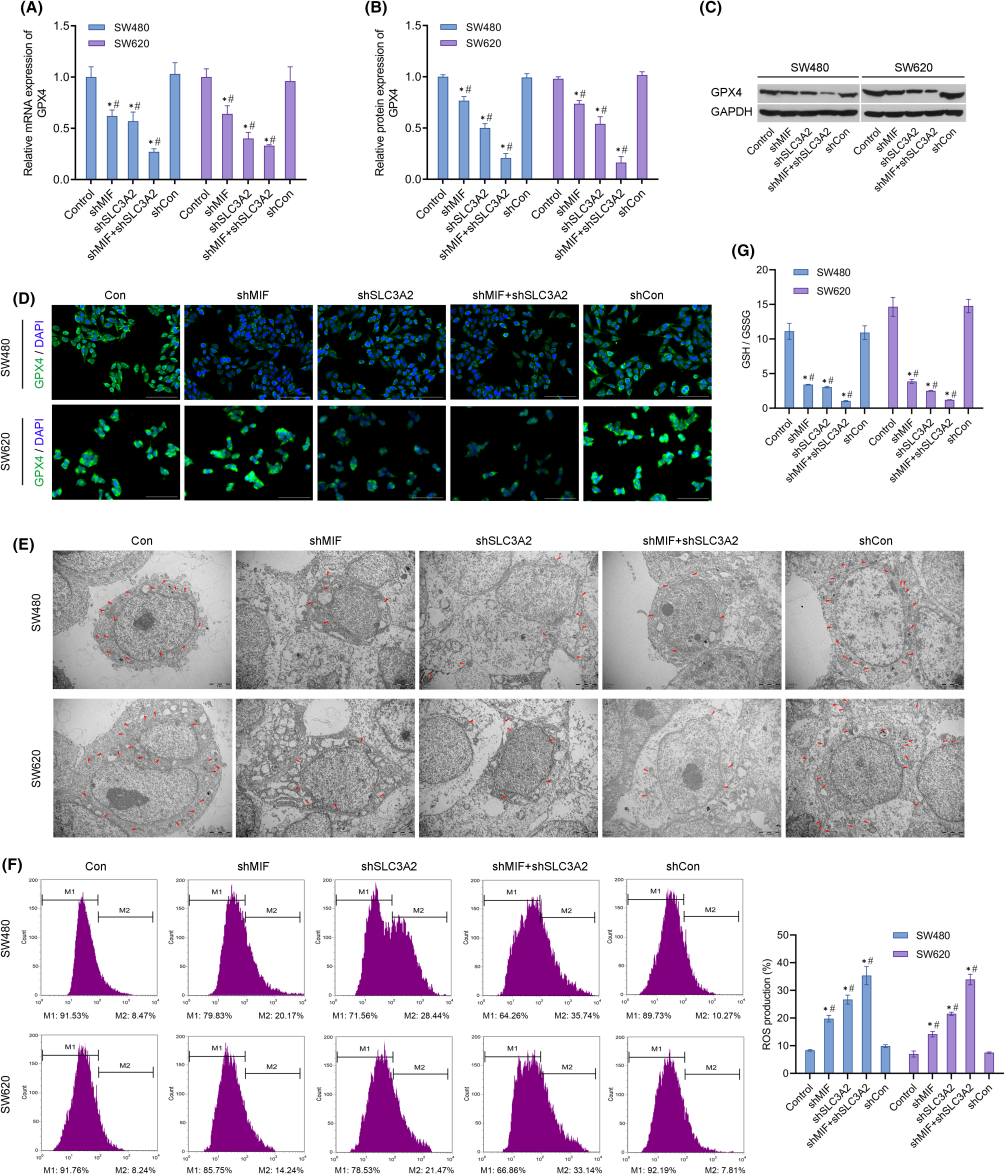
Knockdown of MIF and SLC3A2 promoted iron death in SW480 and SW620 cells. SW480 and SW620 cells were transfected with control shRNA (shCon group), shMIF (shMIF group), shSLC3A2 (shSLC3A2 group) or co‐transfected with shMIF plus shSLC3A2 (shMIF + shSLC3A2 group), respectively. (A) GPX4 mRNA expression in SW480 and SW620 cells. (B) GPX4 protein expression in SW480 and SW620 cells. (C) Western blotting was performed to confirm the levels of GPX4 protein expression in SW480 and SW620 cells. (D) Immunofluorescence assays were performed to detect GPX4 protein expression in SW480 and SW620 cells. (E) The morphology of mitochondria SW480 and SW620 cells was observed by transmission electron microscopy. Mitochondria are shown by red arrows. (F) Detection of ROS in SW480 and SW620 cells by flow cytometry. (G) The ratios of GSH/GSSG in cell lysates from SW480 and SW620 cells were detected by a biochemical method. ‘*’ means *p *< 0.05 when compared with the control group; ‘^#^’ means *p* < 0.05 when compared with the shCon group. GAPDH served as an invariant internal control for calculating fold changes in protein expression

To investigate the effects of MIF and SLC3A2 on iron death, we examined the effects of those two molecules on death‐related proteins and indicators. We found that shMIF and shSLC3A2 significantly decreased the levels of GPX4 mRNA and protein expression (Figure 5A–C). In addition, immunofluorescence assays showed that GPX4 expression was markedly decreased in the shMIF and shSLC3A2 groups, and was even lower in the shMIF+shSLC3A2 group (Figure 5D). More importantly, it was found that shMIF or shSLC3A2 destroyed the structure of mitochondria by making it disordered and fragmented (Figure 5E). As expected, the levels of ROS were significantly increased in the shMIF and shSLC3A2 groups, and were even higher in the co‐transfection group (Figure 5F). In addition, the ratios of GSH/GSSG in the shMIF and shSLC3A2 co‐transfection groups were markedly decreased (Figure 5G).

### 3.6 MIF directly bound to SLC3A2 and regulated the AKT/GSK‐3β signalling pathway

**FIGURE 6:**
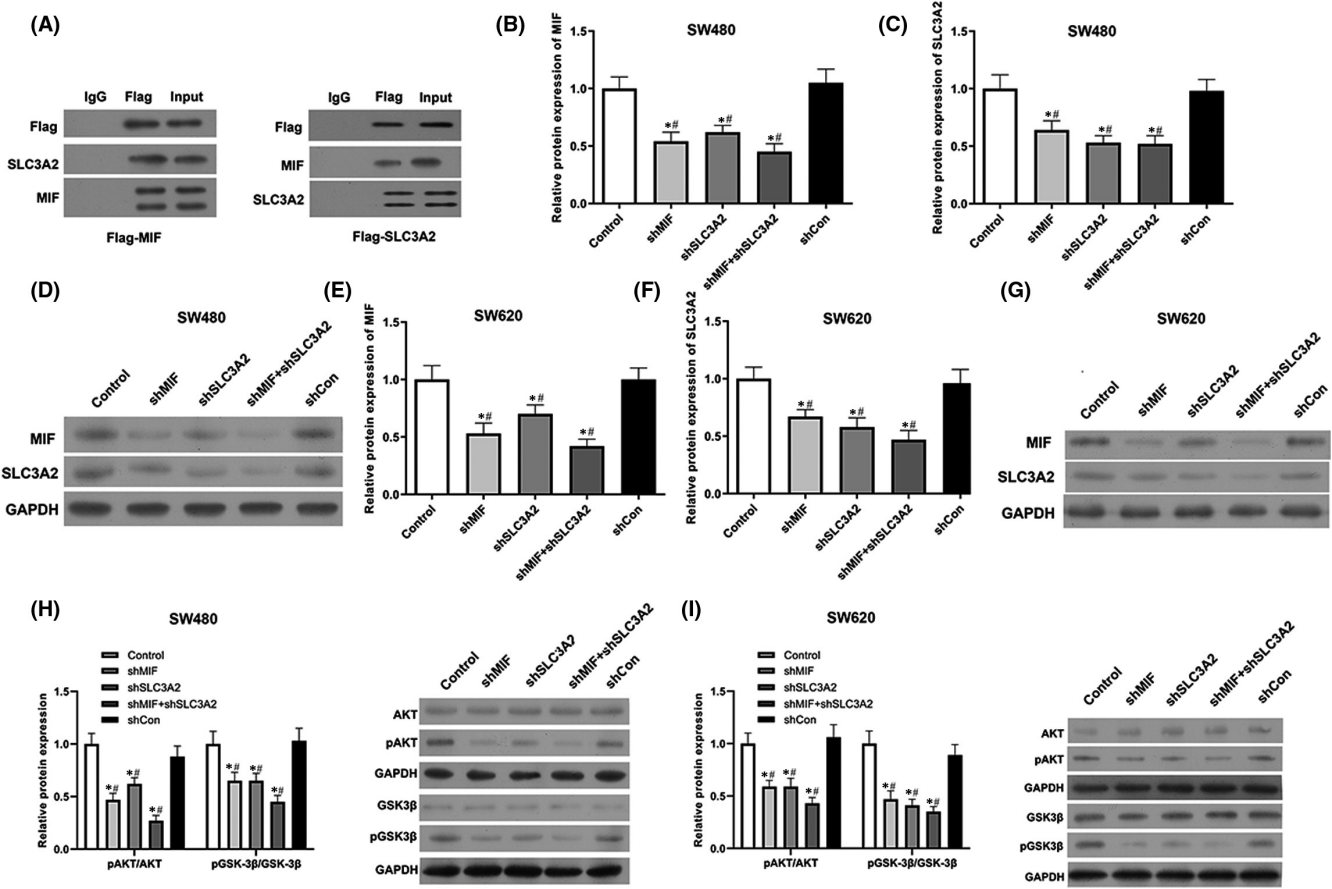
Migration inhibitory factor directly bound to SLC3A2, and regulated the AKT/GSK‐3β signalling pathway. (A) Co‐IP assays were performed to verify the binding of MIF with SLC3A2. (B) MIF protein expression in SW480 cells. (C) SLC3A2 protein expression in SW480 cells. (D) Western blotting was performed to confirm the expression of MIF and SLC3A2 proteins in SW480 cells. (E) MIF protein expression in SW620 cells. (F) SLC3A2 protein expression in SW620 cells. (G) Western blotting was performed to confirm the expression of MIF and SLC3A2 proteins in SW620 cells. (H) pAKT, AKT, pGSK‐3β and GSK‐3β protein expression in SW480 cells. (I) pAKT, AKT, pGSK‐3β and GSK‐3β protein expression in SW620 cells. ‘*’ means *p *< 0.05 when compared with the control group; ‘^#^’ means *p* < 0.05 when compared with the shCon group. GAPDH served as an invariant internal control for calculating fold changes in protein expression

Based on the above results, we speculated that MIF and SLC3A2 might interact in colorectal cancer cells. Co‐immunoprecipitation experiments showed that exogenous or endogenous MIF co‐precipitated with exogenous or endogenous SLC3A2 (Figure 6A). Furthermore, our results showed that shMIF and shSLC3A2 markedly suppressed MIF and SLC3A2 mRNA and protein expression (Figure 6B–D). In addition, the levels of MIF and SLC3A2 expression were down‐regulated in the shMIF and shSLC3A2 groups of SW620 cells, and the lowest expression levels were found in the shMIF + shSLC3A2 group (Figure 6E–G).

Further studies showed that transfection with shMIF or shSLC3A2 could significantly inhibit pAKT/pGSK‐3β expression, and the inhibitory affect was even more significant in the co‐transfection group (Figure 6H,I). These results indicated that MIF regulated the AKT/GSK‐3β pathway by directly binding to SLC3A2.

### 3.7 Knockdown of MIF and SLC3A2 inhibited tumour growth in mice

**FIGURE 7:**
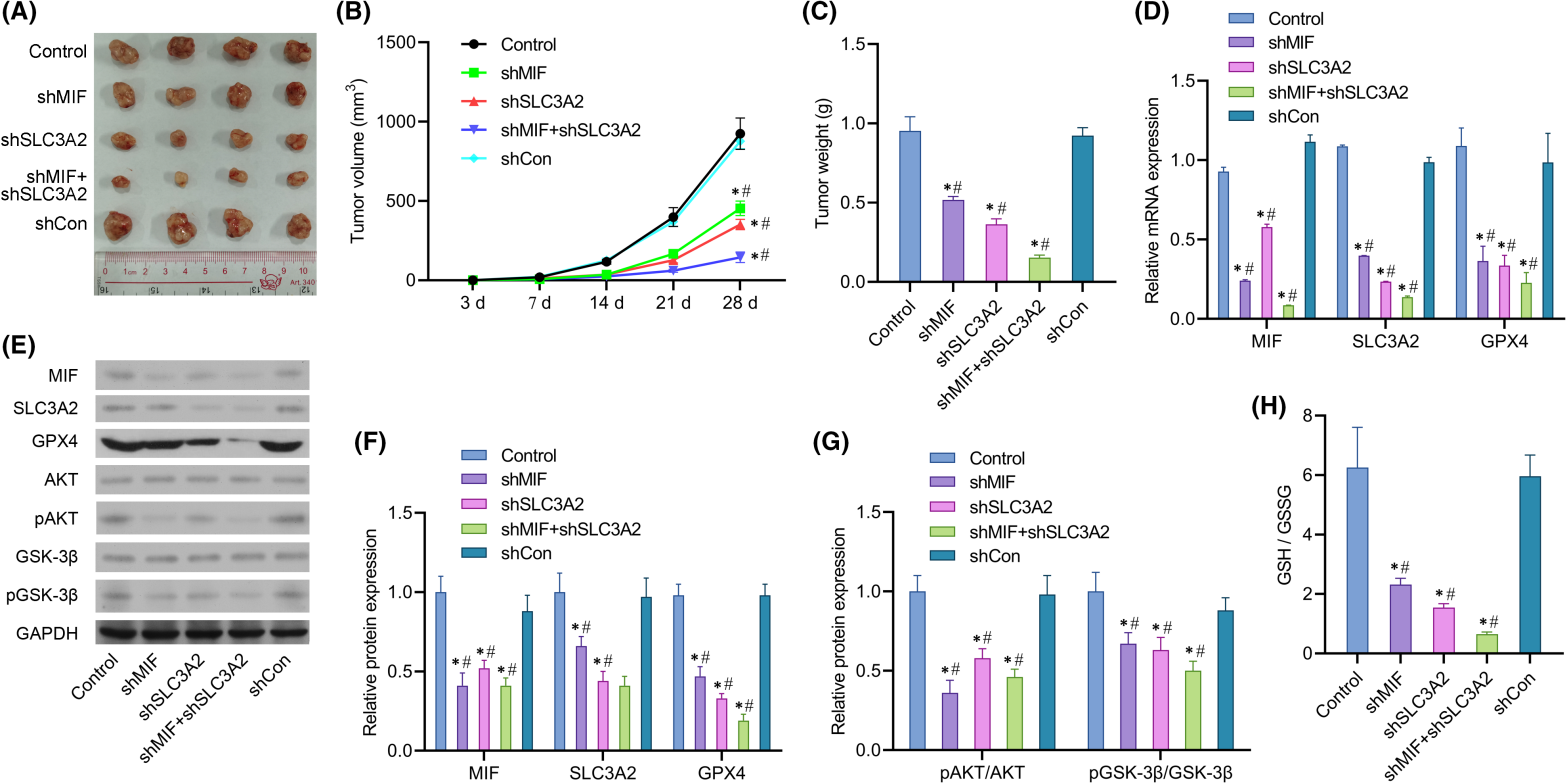
Knockdown of MIF and SLC3A2 inhibited tumour growth and metastasis in mice. Mice were randomly assigned to 5 groups (*n* = 6 per group). A lentiviral vector was constructed and mice in each group were injected with 5 × 10^6^ SW480, SW480‐shMIF, SW480‐shSLC3A2, SW480‐shMIF + shSLC3A2 or SW480‐shCon cells, respectively. (A) Measurements of tumour size in each group of mice. (B) Measurements of tumour volume in each group of mice. (C) Measurements of tumour weight in each group of mice. (D) MIF, SLC3A2 and GPX4 mRNA expression in the tumour tissues of mice in each group. (E) Western blotting was performed to confirm MIF, SLC3A2, GPX4, AKT, pAKT, pGSK‐3β and GSK‐3β protein expression in the tumour tissues of mice in each group. (F) MIF, SLC3A2 and GPX4 protein expression in the tumour tissues of mice in each group. (G) pAKT/AKT and pGSK‐3β/GSK‐3β protein expression in the tumour tissues of mice in each group. (H) The ratio of GSH/GSSG in the tumour tissues of mice in each group. ‘*’ means *p *< 0.05 when compared with the control group; ‘^#^’ means *p* < 0.05 when compared with the shCon group. GAPDH served as an invariant internal control for calculating fold changes in protein expression

We found that inhibition of MIF or SLC3A2 expression in vivo markedly suppressed the growth of tumours, and the inhibitory effect was even more significant in the co‐transfection group (Figure 7A–C).

Further studies showed that knockdown of both MIF and SLC3A2 in vivo significantly inhibited MIF, SLC3A2 and GPX4 mRNA and protein expression (Figure 7D–F). Meanwhile, knockdown of MIF and SLC3A2 suppressed the phosphorylation of AKT and GSK‐3β proteins and activation of the AKT/GSK‐3β signalling pathway (Figure 7E,G). In addition, the ratios of GSH/GSSG in the shMIF and shSLC3A2 co‐transfection groups were markedly lower than those in the control group and single transfection group (Figure 7H).

### 3.8 Knockdown of MIF and SLC3A2 attenuated the histopathological phenotypic characteristics of mouse tumour cells and promoted their apoptosis

**FIGURE 8:**
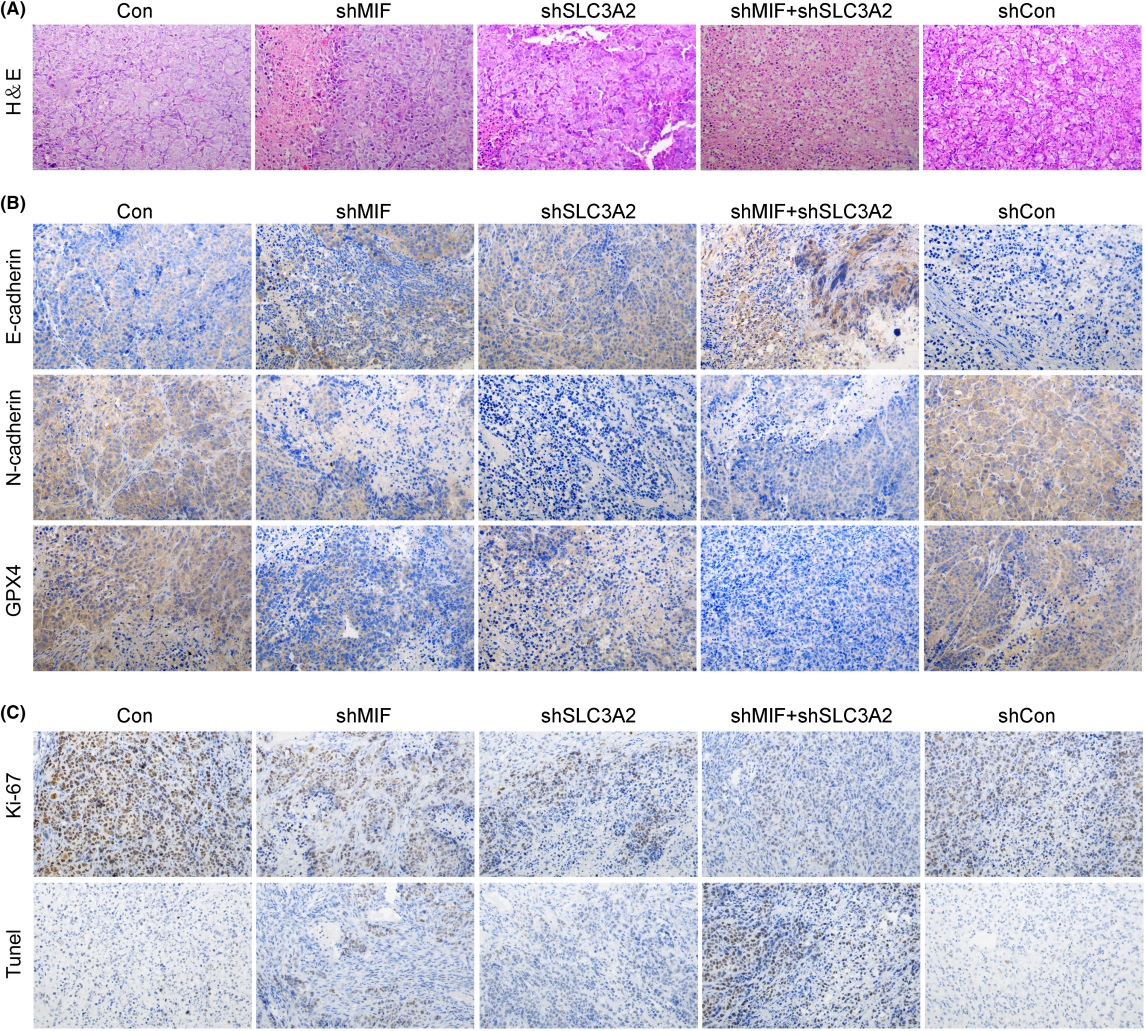
Knockdown of MIF and SLC3A2 attenuated the histopathological phenotypic characteristics of mouse tumour cells and promoted their apoptosis (A) H&E staining was performed to observe the tumour histopathology of mice in each group. (B) E‐cadherin, N‐cadherin, and GPX4 expression were detected by immunofluorescence. (C) Ki‐67 expression was detected by immunohistochemistry. Cell apoptosis in each group was detected by the TUNEL assay

H&E staining results showed that treatment with shMIF and shSLC3A2 significantly attenuated the pathological phenotypic characteristics of mouse tumour tissues to the point that the structural characteristics of the tumour tissues were similar to those of control tissues (Figure 8A). Immunohistochemical analyses revealed that the levels of N‐cadherin and GPX4 expression were down‐regulated in the shMIF and shSLC3A2 groups, while E‐cadherin expression was increased in the shMIF and shSLC3A2 groups (Figure 8B). Additionally, immunohistochemical analyses showed that Ki‐67 expression was decreased in the shMIF and shSLC3A2 groups (Figure 8C). More importantly, inhibition of either MIF or SLC3A2 in vivo significantly promoted the apoptosis of tumour cells, and especially in cells co‐transfected with shMIF and shSLC3A2 (Figure 8C).

## 4 DISCUSSION

Colorectal cancer has high rates of incidence and mortality, and poses a significant threat to human health and life.^25^ In this study, MIF and SLC3A2 were shown to participate in regulating the biological behaviour of colorectal tumour cells and promote the further development of cancer, suggesting that MIF and SLC3A2 might serve as potential biomarkers. In addition, we found that inhibition of MIF and SLC3A2 induced intracellular iron death. It has been reported that GPX4 is the key enzyme which leads to iron death. Therefore, controlling the iron death of cancer cells by inducing GPX4 inactivation might be an effective method for treating cancer.

Pro‐inflammatory mediators, such as MIF, promote the progression of various malignant tumours.^26^ It was found that MIF levels are significantly increased in tumour tissues, indicating that MIF may be an important tumour‐promoting factor.^27^ In this study, we found that knockdown of MIF suppressed the proliferation, migration and invasion of colorectal cancer cells, and inhibited the progression colorectal cancer in vivo. Similarly, Penticuff et al.^28^ found that inhibition of MIF activity significantly attenuated the phenotypic characteristics of cancer cells by regulating ERK or the p53 signalling pathway. In addition, it was reported that MIF expression was increased in samples of advanced cancer tissue and promoted the invasiveness of cancer cells.^29^ Recent evidence suggests that MIF is involved in regulating many key proteins, such as CD74 and HSP90. These reports provide a theoretical basis for developing an anti‐MIF strategy for treating cancer.^30^

In our study, we found that MIF (a soluble factor) could directly bind to SLC3A2 (a membrane‐binding factor) and regulate the function of colorectal cancer cells by promoting SLC3A2 expression. While direct binding between molecules suggests ligand receptor interaction, there may also be other forms of interaction. SLC3A2 is widely expressed in cells and mediates the transmembrane transport of almost all essential amino acids. This transport function ensures the stability of intracellular amino acid levels and plays a key role in maintaining the normal biological function of cells.^31^ Several studies have found that SLC3A2 is overexpressed in many tumours and participates in the malignant transformation of cells.^11^, ^32^, ^33^ It has also been reported that inhibition of SLC3A2 expression significantly reduces the survival rate of ovarian cancer cells treated with cisplatin and promotes cell migration.^34^ Coincidentally, it has been reported that SLC3A2 expression is significantly increased in gastric cancer cell lines and tumour tissues, and those increases are related to serosal invasion.^35^ Our study demonstrated that inhibition SLC3A2 significantly suppressed the proliferation, migration and invasion of colorectal cancer cells in vitro, and inhibited tumour progression in vivo. Furthermore, inhibition of both MIF and SLC3A2 decreased GPX4 expression, promoted ROS production and changed the morphologic characteristics of mitochondria, indicating that MIF and SLC3A2 may be related to iron death, apoptosis, autophagy or necrosis. Therefore, further research is required to fully understand the effects of MIF and SLC3A2.

Previous studies found that the AKT/GSK‐3β pathway is abnormally expressed in different cancers.^36^ As a tumour promoter or inhibitor, AKT/GSK‐3β plays a key role in tumour development.^37^ TWS119 was found to promote the development of breast cancer in mice by regulating GSK‐3β protein expression.^38^ Likewise, our study found that inhibition of MIF suppressed colorectal cancer progression by inhibiting the AKT/GSK‐3β pathway. Inhibition of GSK‐3β can also reduce the proliferation of colorectal cancer cells by down‐regulating NF‐κB and NF‐κB‐mediated gene expression,^39^ which may benefit the clinical outcomes of patients suffering from colorectal cancer.^40^ Our results suggest that downregulation of MIF and SLC3A2 leads to dephosphorylation of Akt and GSK‐3beta; however, the possible mechanism requires further exploration. Furthermore, the mutual interaction and dephosphorylation that occurs between Akt and GSK‐3β may be related to other signalling pathways. For instance, Pan et al.^17^ reported that AKT/GSK‐3β signalling is activated by CD36 in gastric cancer and regulates downstream β‐catenin to promote the further development of gastric cancer. Therefore, inhibition of AKT/GSK‐3β signalling may be a potential strategy for treating human cancer. We will continue to explore the roles and mechanisms of MIF and SLC3A2 in colorectal cancer, so as to provide a basis for targeting those molecules in the clinical treatment of colorectal cancer.

## 5 CONCLUSIONS

In conclusion, our study revealed that MIF participates in the phenotypic regulation of colorectal cancer cells by targeting SLC3A2. Additionally, our in vivo results verified that MIF regulates the biological behaviour of colorectal cancer cells by targeting SLC3A2 and regulating the AKT/GSK‐3β signalling pathway. Therefore, gaining a better understanding of how MIF functions will be useful for developing new therapies for various types of human cancer.

## AUTHOR CONTRIBUTIONS

**Guan Huang:** Data curation (equal); Formal analysis (equal); Investigation (equal); Writing – original draft (equal). **Lili Ma:** Data curation (supporting); Project administration (lead); Supervision (supporting). **Lan Shen:** Data curation (supporting); Investigation (supporting). **Yan Lei:** Investigation (equal); Methodology (equal); Project administration (equal). **Lili Guo:** Conceptualization (equal); Methodology (supporting); Writing – original draft (supporting). **Yongjian Deng:** Investigation (equal); Methodology (equal); Writing – original draft (supporting); Writing – review & editing (supporting). **Yanqing Ding:** Formal analysis (equal); Funding acquisition (equal); Methodology (equal); Writing – review & editing (equal).

## CONFLICT OF INTEREST

All authors declare having no conflict of interest.

## Data Availability

The datasets generated and/or analysed during the present study are available from the corresponding author on reasonable request.
